# Primary pancreatic hydatid cyst—a rare case report with review of literature

**DOI:** 10.1093/jscr/rjaf150

**Published:** 2025-03-19

**Authors:** Areeba Khursheed, Shahbaz Habib Faridi, Syed Hasan Harris, Bushra Siddiqui, Mohammad Nafees Ahmad

**Affiliations:** Department of Surgery, JN Medical College, Aligarh Muslim University, Aligarh, Uttar Pradesh 202002, India; Department of Surgery, JN Medical College, Aligarh Muslim University, Aligarh, Uttar Pradesh 202002, India; Department of Surgery, JN Medical College, Aligarh Muslim University, Aligarh, Uttar Pradesh 202002, India; Department of Pathology, JN Medical College, Aligarh Muslim University, Aligarh, Uttar Pradesh 202002, India; Department of Surgery, JN Medical College, Aligarh Muslim University, Aligarh, Uttar Pradesh 202002, India

**Keywords:** hydatid disease, pancreato-biliary, cystectomy, omentoplasty

## Abstract

Hydatid disease is caused by the larval stage of *Echinococcus granulosus*. It most commonly affects the liver and lungs. Pancreatic hydatid cyst is very rare with incidence of 0.14%–2%. Presenting symptoms vary depending on the location and size ranging from mild non-specific symptoms to less commonly encountered serious pancreato-biliary complications. Due to non-specific symptoms, overlapping imaging features in the early stages and low index of suspicion, the preoperative diagnosis remains a challenge. We present the case of 23-year-old Asian male who presented with complaints of nausea, abdominal pain and vague abdominal lump for 6 months. The patient vague complaints and initial radiological investigations underscore the importance of considering pancreatic hydatid cyst as the diagnosis. Magnetic resonance imaging (MRI) whole abdomen revealed a well defined thick walled cystic lesion in relation to the head of pancreas with undulating membrane noted within the cyst (detached endocyst). The patient underwent open partial cystectomy with omentoplasty along with anti-helminthic therapy. Histopathological findings revealed lamellated membrane with an inner, degenerated germinal layer comprising degenerated protoscolices with hooklets suggestive of hydatid cyst.

## Introduction

Hydatid disease is a parasitic infection which is prevalent primarily in areas where livestock farming and agriculture are widespread [1]. Four species of *Echinococcus* are responsible for hydatid disease in humans [2]. Of these, *Echinococcus granulosus* is the most frequently encountered. In its lifecycle, dogs are the definitive hosts, with sheep and goats serving as intermediate host. Humans are accidental, dead end host.

The liver is the primary site of infection followed by lung. Pancreatic involvement is extremely rare. It accounts for <2% of systemic cases with only 33 cases documented in literature since 2011 [3]. Among the pancreas, the head is most commonly affected, followed by the body and tail [4].

In this case, we present a rare instance of a primary pancreatic hydatid cyst (PHC) that was misidentified as a pancreatic pseudocyst and subsequent management.

## Case report

A 23 year old male presented with a 6-month history of epigastric pain and nausea. The pain was gradual, continuous, non-progressive, and mild in intensity. The pain radiated to the back and was dull aching in character with no aggravating or relieving factors. There were no other significant symptoms. On physical examination, a firm 7 × 7 cm lump was palpated in the epigastric region and left hypochondrium. It was spherical with ill-defined margins, smooth surface and did not move with respiration. The mass was not contiguous with liver dullness. When the patient was positioned in the knee-elbow position the lump did not fall forward suggesting retroperitoneal location.

Blood tests were unremarkable and serum amylase (103 U/L) and lipase (165 U/L) were within normal limits ruling out acute pancreatitis. An abdominal ultrasound identified a cystic lesion in the head of the pancreas measuring 7 × 8 cm. Contrast enhanced computed tomography (CECT) whole abdomen revealed a unilocular, thick walled, hypodense cyst (7.5 × 8.2 × 10 cm) near the head and uncinate process of pancreas. The cyst displaced the antrum of the stomach, transverse colon, first and second part of the duodenum, suggestive of a pancreatic pseudocyst. However, the absence of trauma or pancreatitis made this diagnosis unlikely. MRI whole abdomen revealed a well defined, thick walled cyst with a characteristic undulating membrane suggesting hydatid cyst (Fig. 2). Echinococcal serology was positive, with IgG (16 U/ml) and IgM (20 U/ml) confirming the diagnosis of hydatid cyst.

**Figure 1:**
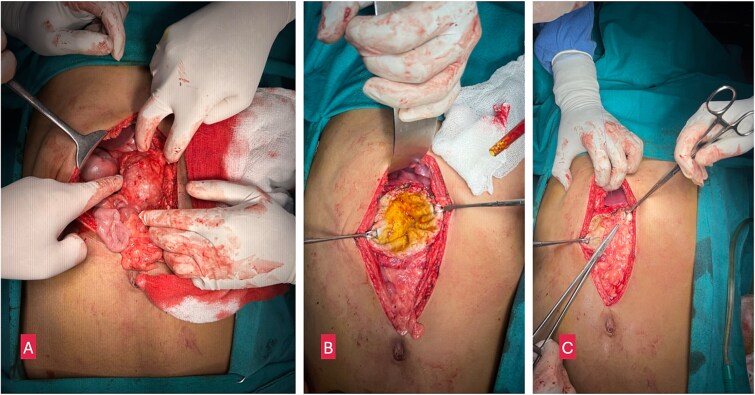
(A) Intraoperative image showing a large cystic lesion in the head of pancreas. (B) Opened up cyst after partial pericystectomy. (C) Omentoplasty.

The patient was diagnosed with a primary isolated PHC and underwent open partial pericystectomy and omentoplasty, along with perioperative albendazole therapy (15 mg/kg/day). During surgery, a large, thick-walled cyst (7 × 8 cm) was found in the head and uncinate process of the pancreas, displacing the duodenum and bulging through the transverse mesocolon (Fig. 1). The cyst was isolated using 10% povidone-iodine-soaked gauze, and decompression was performed by needle aspiration. A scolicidal agent (10% povidone-iodine) was used to sterilize the cavity before partial cystectomy (deroofing) was performed.

**Figure 2:**
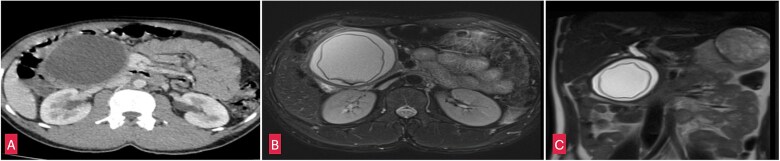
(A) Coronal section of CECT abdomen showing a thick walled, well defined round to oval cystic lesion ~7.5 × 8 × 10 cm in the head and uncinate process of pancreas. (B) Coronal section of MRI-magnetic retrograde cholangio-pancreatography (MRCP) abdomen showing a well defined thick walled cystic lesion in the head and uncinate process of the pancreas with undulating membrane noted within the cyst. (C) Saggital section of MRI-MRCP abdomen with similar findings as above.

**Figure 3:**
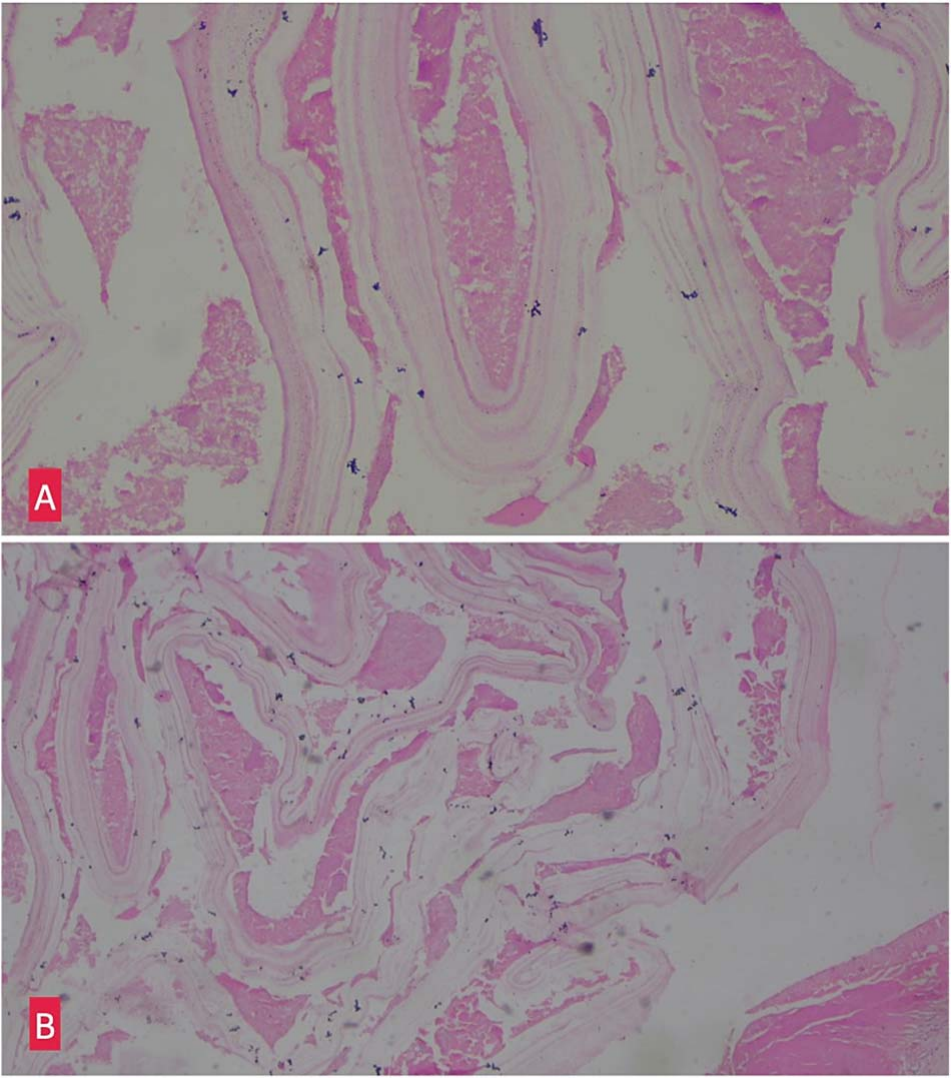
(A) Hemotoxylin and eosin ×10: Section shows lamellated appearance of hydatid cyst. (B) Hemotoxylin and eosin ×40: Section shows lamellated appearance of hydatid cyst.

Histopathological examination of the excised cyst wall confirmed the diagnosis, showing a lamellated membrane (Fig. 3).

Postoperative period was uneventful and the patient was discharged on oral albendazole therapy for 8 weeks. He had made a full recovery and is doing well on follow-up visits.

## Discussion

Humans do not play a direct biological role in the life cycle of *E. granulosus* and are accidentally infected by ingesting eggs found in the feces of infected dogs. Upon ingestion, the eggs pass through the intestinal wall, enter the portal circulation, and ultimately lodge in liver [5]. The most common mechanism of spread being hematogenous dissemination [6]. Other pathways include through the biliary system, lymphatic spread, or direct migration [6].

The growth of these cysts is slow, averaging 0.3–2 cm per year [7], and many patients remain asymptomatic for years.

Hydatid cysts in the head of the pancreas may present with obstructive jaundice or acute pancreatitis. Cysts in the body of the pancreas are usually silent until they grow large enough to cause abdominal distension, nausea, vomiting, and abdominal pain. On rarer occasions, cysts located in the tail may lead to splenomegaly or portal hypertension [7].

Ultrasonography is a non-invasive and cost-effective tool that is often the first choice, but its utility is limited in pancreatic cysts due to the retroperitoneal location of the pancreas and the interference from bowel gas. CT scan is more effective for determining cyst size, location, and its relationship to the pancreatic and biliary systems. MRI is particularly useful for evaluating the cyst's relationship with the bile ducts and pancreas [8].

Surgical intervention remains the primary treatment for PHCs, and the approach depends on the cyst's location.

## Conclusion

Primary pancreatic hydatid cyst is a rare entity that can masquerade as pseudocyst or cystic neoplasm making the diagnosis challenging. The prognosis is usually favourable when appropriately managed. Surgical intervention combined with albendazole remains the main stay of treatment.

## Data Availability

The information generated during the case report is included in this published article and is available for review.
